# Health and education: service providers in partnership to improve mental health

**DOI:** 10.1186/1752-4458-6-19

**Published:** 2012-09-20

**Authors:** Valsamma Eapen, Lily Lee, Craig Austin

**Affiliations:** 1 Academic unit of Child Psychiatry South West Sydney (AUCS), University of New South Wales, ICAMHS, Mental Health Centre, L1 Liverpool Hospital, Elizabeth Street, Liverpool NSW 2170, Sydney, Australia; 2 Liverpool-Fairfield Child and Adolescent Mental Health Service, 53-65 Mitchell Street, Carramar, Sydney, Australia; 3 Glenfield Park, Sydney, Australia

**Keywords:** Access to mental health services, Children, Adolescents

## Abstract

**Background:**

Children and adolescents from complex or disadvantaged backgrounds and multiple needs often are reluctant to seek help and this is particularly relevant in the context of mental health difficulties. Further, the complexity of the health system can be overwhelming to the family who are likely to be chaotic and less able to seek help. The current project piloted an integrated service delivery model involving a child psychiatry service and the department of education to promote access to mental health assessment and intervention to young people attending special education schools in Sydney, Australia.

**Findings and conclusion:**

The project allowed improved access to mental health services for a group of young people who would otherwise not have sought help through traditional referral pathways. Our findings support strategies to promote the social milieu of schools as a way of achieving better mental health and learning outcomes.

## Introduction

Despite many years of attempt to de-stigmatize the concept of ‘mental health’ by various health organisations and governments, accessing mental health services is still perceived by many parents and their children as a daunting task. For example a study of first-time primary care giver’s experience of accessing first episode psychosis services revealed that caregivers often encountered service-focused and carer-focused barriers when initially accessing services. However, acquired knowledge, experience with services and caregiver assertiveness was noted to enhance access on subsequent occasions. Hence programs aimed at engaging the families coupled with providing information might empower them to better access mental health services. One potential but currently neglected focus for health intervention is the school’s social milieu. [1-3] There is some evidence that the school’s social atmosphere affects patterns of substance use, antisocial and disruptive behaviours as well as how well students learn. Advocates of health promotion in the school context have argued that addressing organizational processes and social relationships are likely to be effective in bringing about behavioural change. [4-6] Despite the attractiveness of utilizing the resources and context of school for facilitating health interventions, relatively few strategies have been tested that use this approach. [7-9].

In 2008, three New South Wales government special schools located at the “Hill Top”, Glenfield, and the Child and Adolescent Mental Health Service of the Sydney South West Area Health Service (now called the South Western Sydney Local Health District) started a project to overcome the barrier in accessing health services and with a view to provide a holistic service through collaboration between education and health departments.

Support for the project was obtained through discussions between the senior management of the Health and Education departments in response to a desire to develop better partnerships.

### Research focus and objectives

a) Developing a framework for collaboration between Health and Education departments in the provision of mental health assessments and recommendations

b) Upskilling teachers and school staff on detection of mental health issues through consultation and training opportunities.

c) Facilitating access to young people and improving engagement of families to early intervention and therapeutic management of significant behaviour and emotional problems.

d) In-service training for school personnel to develop skills and knowledge in mental health, to equip them better in managing challenging behaviours in the classroom.

The program was evaluated using a qualitative approach and involved school staff who made the referrals and focus group reviews with senior management. For the clinical outcome of the young people who were assessed through the program, the Child Behaviour Checklist (CBCL- Parent and teacher scale) [10] was used.

### The Schools for Specific Purposes (SSP)

The three government schools involved in this Hill Top project are Ajuga, Campbell House and Glenfield Park SSP’s. All three schools have higher ratio of teachers to students (1:7) compared to mainstream school. Students are placed at these schools when they are unable to continue their education at the mainstream school due to their mental health and/or challenging behaviours. Ajuga and Glenfield Park SSP cater for students from kindergarten to high school years, with a view of integrating students back into their mainstream school. All students at Glenfield SSP have mild intellectual disability as a co-morbid disability. Campbell House caters for only high school students and the focus is on integration to higher education or work. Although the schools are located in the Campbelltown LGA (south region of South Western Sydney), their students come from a broader geographical area of Sydney.

### Child and Adolescent Mental Health Service

The project operated as an outreach service of the Child and Adolescent Mental Health Service (CAMHS) covering the local government areas of Fairfield and Liverpool in South Western Sydney. This service has a strong family focus in their intervention, and a secondary service which provides services for children and adolescents with moderate to severe mental health problems following referrals from professionals (e.g. school counsellors, doctors, etc.).

### The Hill Top Project

The Project was coordinated by a clinician from the mental health services and a district guidance officer (DGO and senior school counsellor) based at one of the three schools. The project consists of three components: the consultation clinic, training for teachers, and seminars on mental health topics for parents of students attending these schools.

For the consultation clinic, the CAMHS clinician and the DGO organise referrals and the clinic is conducted once a month during school term. The team usually include a child & adolescent psychiatrist and a clinician from CAMHS. When the school personnel raise concerns about a student, the school counsellor prepares a referral report with information on presenting mental health problems, family background and obtains consent from the parents/carers for a referral to the consultation clinic.

### The consultation clinic typically consists of three stages

(1) An initial consultation with school personnel that includes the DGO, school counsellor, school principal and classroom teacher(s) who have direct involvement with the student. The purpose here is to provide a triage to determine the severity of the mental health impacting on the student’s learning and day to day function, provide recommendation and support for the school and to assess the need for further psychiatric or other interventions.

(2) If after the initial consultation, it is determined that the student would benefit from a clinical mental health assessment, this is provided either at the school itself or at the CAMHS service depending on the family’s level of acceptance of mental health intervention and availability of transport. If the student lives outside of the CAMHS catchment area, the CAMHS clinician will facilitate a referral to an appropriate child and adolescent mental health service.

(3) The cases are reviewed at least once after initial consultation to ensure that recommended actions have been completed.

### Training for teachers

The CAMHS psychiatrist and clinicians provide a minimum of two training sessions per year at the school that is attended by around 30 staff members. School principals decide the mental health topics that are most relevant to their teachers. The training usually involves a 90 minute session covering both the theoretical underpinnings and the practical skills as relevant to the topic. The objective is to increase awareness and knowledge and thereby up-skill teachers to better support the learning needs of students who have mental health problems. Topics covered in training sessions have included management of young people with history of trauma, clinical presentations in autism spectrum disorder, management of challenging behaviours in the class room etc.

### Seminars for parents

The school holds regular parents meetings to provide support to parents of students attending the schools. Schools also can request psycho-education presentation as deemed appropriate.

## Results

Till date a total of 32 clinics were held as part of the project where initial consultation with the school personnel occurred and in some cases the family and the patient were also involved. Of the 28 cases (25 males and 3 females) that progressed to further evaluations, 5 had family interviews at the school clinic and 5 were followed up through the CAMHS services. The rest were subsequently followed up by school, referred to paediatricians or other mental health services when the students were from outside the catchment area. Of the 10 students who received a comprehensive assessment, all had clinical features that warranted a diagnosis as per the Diagnostic and Statistical Manual -4^th^ Edition [DSM-IV] and they received appropriate interventions. These included Conduct Disorder, Oppositional Defiant Disorder, Attention Deficit Hyperactivity Disorder, Depressive Disorder, Anxiety Disorder, Bipolar disorder, Dissociative disorder and Autism Spectrum Disorder.

At the 6 monthly and end of the year focus group review, teachers who made the referrals reported that they found it valuable to have advice from the mental health professionals on the management of students in crisis. Feedback from the parents who attended the CAMHS service following the referral through this project had been positive and they articulated that they were better able to access coordinated services by health and education, which would not have been possible otherwise. School professionals who made the referrals completed a semistructured questionnaire about their experience of the program in which they agreed that the collaborative efforts had helped to provide a comprehensive approach to management of mental health issues in schools. There was 100% agreement from staff who made the referrals that the project “definitely made it easier to engage families” and that “parents and students have been more likely to follow through with appointments” when the referral was made through this project. While some families had been reluctant in the past to attend appointments at the heath facility, the opportunity for consultation at the school made it possible to engage the family and for the young person to receive a mental health assessment. Other themes included the feedback from teaching staff that the project helped improve their knowledge and understanding of mental health issues as well as the confidence to deal with these issues.

### Case vignette

X, an adolescent male student was referred for significant problems in attention as well as disruptive behaviours in class (e.g., making bird noises, leaving classroom, etc.). His parent was reluctant to seek specialist help to assess him for mental health intervention. However, with encouragement from his teacher and school counsellor, his parent agreed to meet with the mental health staff at the consultation clinic. He was then diagnosed with Tourette Syndrome and prescribed medication. His behaviour improved significantly both at school and at home as assessed by the Achenbach School-Age assessment Teacher’s Report checklist, Health of the Nation Outcome Scales for Children and Adolescents (HoNOSCA) [11] and Clinical Global Assessment Scale in October 2010 and May 2012 (Table 1).He was able to pursue his career goal through training at a tertiary institution while continuing his enrolment at the special school on a part time basis.

**Table 1:** Pre- and Post evaluation of case X

	**Pre test (October 2010)**	**Post test (May 2012)**
Anxious Depressed	Normal	Normal
Withdrawn Dep	Normal	Normal
Somatic complaints	Normal	Normal
Social Problems	High	Borderline
Thought Problems	Clinical range	Borderline/Normal
Attention Problems	Clinical range	Normal
Delinquent Behaviour	Normal	Normal
Aggressive behaviour	High	Normal
Other Problem in total	Normal	Normal
Internal	Clinical Range	Normal
External	Clinical Range	Borderline
Total	High	Borderline

### Pathways to Care

Facilitating “pathways to care” was seen as an important outcome of this initiative with three key elements addressed namely:

A. Better access to mental health services

B. Coordinated referral to the appropriate mental health service that matched the individual needs

C. Collaboration and consultation to improve:

a. Knowledge about mental health presentations

b. Skills and ability to manage mental health problems

c. Systematic changes in the way mental health issues are addressed in the school environment

## Discussion

The key elements of the intervention included process-feedback on the social context, creation of a coordinating structure (i.e. the action team), and ongoing consultation. The process of coordinated health promotional work provided a means for nesting a health agenda within a school’s policy and practice framework. The result was the development of interventions tailored to the needs of particular students that was suitable for the school and home environments. Until recently mental health has been seen as primarily the domain of health specialists, and not the concern of teachers, parents and police [12]. However, with the increase in the number of students with mental health problems in schools and particularly in the special education settings, and given the lack of specific preservice preparation in the area of mental health for teachers [13], they often remain unprepared to recognize and/or intervene in mental health issues confronting today's teachers. Although there remains much to learn about the wider application of this particular approach, our findings support strategies to promote the social milieu of schools as a way of achieving better health and learning outcomes [6,7]. While there are other examples of projects involving clinical assessment of young people with intellectual disability in the school setting, the Hill Top project described here is distinct in that the focus is the health promoting “whole-school approach” [14] where staff, parents and young people are all active participants in this program. This project is a rare example of a unique partnership between Area Mental Health Services and the Department of Education where collaborative clinical service is complemented by teacher training and parental involvement. Having established this strong partnership, out next aim is to maintain and extend this initiative to other schools in the district in order to provide similar opportunities.

## Conclusion

The Hills Top project facilitated collaboration between health and education departments that allowed improved access to health services for a group of young people who would otherwise not have sought help through traditional referral pathways.

## Competing interests

The authors declare that they have no competing interest.

## Authors' contributions

VE made substantial contributions to conception and design of the project as well as being involved in the delivery of the service, data gathering and finalising the manuscript. LL was involved in project planning, data analysis and in manuscript preparation. CA contributed to project co-ordination at the site and data collection. All authors read and approved the final version of the manuscript for publication.
